# Excess cement and peri‐implant disease: A cross‐sectional clinical endoscopic study

**DOI:** 10.1002/JPER.24-0510

**Published:** 2025-01-15

**Authors:** Marco Montevecchi, Leoluca Valeriani, Maria Francesca Salvadori, Martina Stefanini, Giovanni Zucchelli

**Affiliations:** ^1^ Department of Biomedical and Neuromotor Sciences School of Dentistry – Division of Periodontology and Implantology Alma Mater Studiorum – University of Bologna Bologna Italy

**Keywords:** dental implants, endoscopy, excess cement, implant prosthesis, peri‐implant disease, risk factor

## Abstract

**Background:**

Crown cementation is a common technique for implant‐supported prosthodontics. However, for possible slipping of the cement below the mucosal margin, its thorough removal poses some issues. The objective of this study was to evaluate the presence of submucosal cement residues in patients with peri‐implant disease by endoscopic visualization and to investigate the potential correlation between the pathological scenario and the spatial position of cement residues.

**Methods:**

The study sample comprised 46 consecutive patients exhibiting clinical and radiographic signs of peri‐implant disease around cement‐retained crowns. When at first visit bleeding on probing was the only pathological sign, the area was debrided and then treated with antiseptic agents for 30 days. Only those patients for whom bleeding was still found at re‐evaluation were included in the study. All implants were therefore endoscopically evaluated to identify possible submucosal cement residues. For all implants showing residual cement, the spatial position of the residual cement was recorded with respect to predefined points.

**Results:**

Cement residues were detected in 80.4% of the patients and were predominantly located in the vestibular and lingual areas (88%). Analysis of the distances between the apical portion of the residues and anatomical landmarks revealed significant differences between mucositis and peri‐implantitis patients (*p* = 0.001). In cases of peri‐implantitis, the cement residue was more apically located than in mucositis.

**Conclusion:**

The presence of cement residue was associated with peri‐implant disorders in the majority of patients. A potential correlation between the position of residues and the peri‐implant disease scenario is here suggested.

**Plain Language Summary:**

A common method for fixing the crown on the dental implant is the use of specific cements. However, these products can slip below the gum line resulting in difficulties in their removal, which could cause peri‐implant disease. This study aimed to evaluate the presence and location of residual cement hidden under the gums in patients with peri‐implant disease, characterized by symptoms such as bleeding gums and, in some cases, bone loss around the dental implant. Using a small fiber‐optic camera (endoscope), 46 patients were examined for any cement residue under the gums. The study showed that more than 80% of patients had cement residues, especially in the areas facing the cheeks and tongue. It was also found that in patients with a more severe form of inflammation (peri‐implantitis), the cement was placed deeper than in those with a milder form (mucositis). This confirms that residual cement is common in patients with peri‐implant disease, but also suggests that its location could influence the severity of the pathological manifestation. This finding highlights the importance of carefully removing all cement to prevent such complications.

## 1 INTRODUCTION

Cemented prostheses are one of the options for implant‐supported rehabilitation. In this technique, the prosthetic crown is fixed to the implant by direct cementation to the abutment, which is already anchored to the implant.

The main limitation of this procedure is the difficulty of controlling the amount of dental cement that can slip below the mucosal margin (MM) during the cementation phase, thus making its full removal particularly difficult.^1^

The depth of the restoration–abutment interface with respect to the MM can undoubtedly influence the ability to remove cement. This aspect is particularly relevant for aesthetic areas, where the interface is frequently positioned even more than 3 mm below the gingival margin to conceal the metal prosthetic components and obtain aesthetically pleasing emergence profiles.^2^, ^3^ In these anatomical areas, frequent deep mucosal tunnels and therefore greater difficulties in removing excess cement are more likely.^4^, ^5^

Several studies have hypothesized that there is a correlation between excess cement and the incidence of peri‐implant diseases. However, its potential role in peri‐implant disease development is not yet clear. There are two main etiopathogenic hypotheses: one direct and one indirect.

In the direct route, both a chemical and a mechanical role can be hypothesized. Based on the relative biocompatibility of these products, cement could be considered an “allergen” capable of first evoking sensitization and then triggering a local reaction.^6^

The encumbrance of the surface by the residual material could instead induce a foreign body reaction capable of releasing proinflammatory cytokines involved in the bone resorption process.^7^

The indirect route, on the other hand, proposes a bacterial etiology in which cement residues become the cause of adhesion and retention of bacteria, with consequent formation and support of pathogenic biofilms.^8^, ^9^

Tatullo et al., when analyzing failed implants that had cement residues, detected a foreign body reaction amplified by the bacterial component adhering to the residues themselves—findings suggesting that both routes are possible and very likely interact.^10^

In this context, it can be speculated that a patient with a previous history of periodontal disease with the probable presence of individual predisposing factors could be more exposed to peri‐implant diseases induced by cement residues.^11^, ^12^

The main clinical‐instrumental investigation techniques for checking any cement residue at the submucosal level are radiographic examination and clinical exploration of the area with explorer probes. Radiographic examination is a widespread method, although an analysis of the literature shows that it is not a reliable procedure for detecting cement residues. In a retrospective study, as reported by Linkevicius et al., residual cement was radiographically detectable at the mesial level in only 7.5% (4/53) of the patients and distally in 11.3% (6/53) of the patients.^13^ The detectability also depends on the radiographic density of the product used. Cements for implant prostheses generally have a low radiodensity, which makes them difficult to detect radiographically.^14^, ^15^ Finally, an obvious and concrete limitation is that the radiographic examination does not allow the detection of cement residues placed in a buccal or lingual position.

On the other hand, manual probing with an explorer is generally considered an inaccurate diagnostic tool because it strictly depends on the operator's tactile sensitivity and clinical accessibility to the site.

Osborn et al., comparing the detection of subgingival calculus with two methodologies, demonstrated that a dental endoscope is much more efficient than a conventional explorer.^16^ This finding suggested that the visual component positively and significantly contributes to the detection of subgingival calculus. To date, the dental endoscope is the only minimally invasive clinical instrument capable of allowing a direct view of both the hard and soft tissues below the gingival margin.

In a clinical study using an endoscope, Wilson et al. reported that 80% of implants with a cemented prosthesis and signs of peri‐implant disease had submarginal cement remnants and that their location was primarily buccal or lingual.^8^ However, this interesting study does not provide information on the possible pathogenic role of cement in relation to its spatial location below the gingival margin.

The growing evidence on the relationship between excess cement and peri‐implant diseases suggests further investigations on this topic, an aspect that was also clearly underlined during the 2017 World Workshop, defining it as an “Area of Future Research.” ^17^

Hence, the primary aim of this cross‐sectional clinical study was to evaluate the presence of cement residues at the submucosal level in patients with peri‐implant disease using a dental endoscope. The secondary aim was to investigate the possible correlation between the severity of the pathological picture and the more apical extension of the cement residues with respect to the implant‐prosthetic unit.

## 2 MATERIALS AND METHODS

### 2.1 Study population

This cross‐sectional study was conducted on patients attending the Division of Periodontology and Implantology, Dental School, “Alma Mater Studiorum” University of Bologna, Italy, from October 2018 to July 2019. The study protocol was previously approved by the ethics committee (Comitato Etico di Area Vasta Emilia Centro della Regione Emilia‐Romagna prot. no. 120916 del 8/10/2018) and registered at ClinicalTrials.gov as NCT05945836. The study was conducted following the principles of the Declaration of Helsinki for research involving human subjects. Prior to thier inclusion, all participants provided written informed consent.

All adult subjects (≥18 years) who presented at least one cemented single‐implant restoration with clinical signs of peri‐implant disease were consecutively selected for the study.

To enter the study, subjects with clinical signs of attachment loss had to have already completed active periodontal therapy (healthy but reduced periodontium) before implant rehabilitation.

Individuals with neuromotor pathologies or morphological‐dysfunctional alterations capable of interfering with the correct use of the dental endoscope were excluded.

In the case of clinical and radiographic diagnosis of peri‐implantitis, the subject directly entered the study sample. In the case of peri‐implant mucositis, an accurate debridement session was performed with home antiseptic irrigation of 0.2% chlorhexidine twice a day for 10 days. Only if the pathological picture was still present at the 1‐month re‐evaluation was the patient enrolled in the study.

Following the guidelines of the World Workshop on the Classification of Periodontal and Peri‐implant Diseases and Conditions 2017, the same operator (M.M.) performed the initial clinical and radiographic diagnosis, as well as the re‐evaluations of the cases of peri‐implant mucositis after the debridement session.^18^

The following data were collected for each subject: sex, age, periodontal history, smoking habit, cause of implant rehabilitation (trauma, caries, periodontitis, agenesis, other), and time elapsed between prosthetic rehabilitation and pathological picture.

Clinical observation of the submarginal portion of the implant was performed with the use of a dental endoscope (DV2, Dental View, Irvine, California, USA). The analysis was performed atraumatically, and anesthetic infiltration was administered only at the request of the patient.

This clinical analysis was performed by the same periodontist with consolidated experience in periodontal endoscopy (M.M.). The interpretation of the endoscopic images as well as the recording of the clinical data derived from them were jointly discussed between two distinct operators (M.M., M.F.S.).

During the endoscopic examination, once a deposit had been identified, its consistency and adhesion were checked by means of a water jet or probe. For solid deposits, a differential analysis between calculus and cement was carried out. As previously described by Wilson et al. (2009), cement was identified by its characteristic white reflectance, while subgingival calculus was yellow or brown depending on the lighting intensity.^8^

If the deposit was recognized as a cement residue, its presence was recorded, and the following data were collected:

‐ Anatomical location of the residual cement (mesial, distal, lingual/palatal, or buccal)
‐ Distance between the mucosal margin and the prosthetic closing margin at the site of the residual cement: MM–PM = mucosal margin–prosthetic margin
‐ Distance between the prosthetic closing margin and the implant platform at the residual cement site: PM–IP = prosthetic margin–implant platform
‐ Distance between the most apical portion of the cement residue and the implant platform: AC–IP = apical cement–implant platform

**FIGURE 1:**
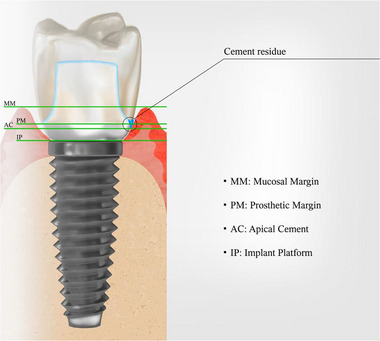
Section of cement‐retained implant‐supported prosthesis showing reference points used to calculate distances. Cement residue is represented in blue. AC, apical cement; IP, implant platform; MM, mucosal margin; PM, prosthetic margin.

An illustration of the different distances is presented in Figure 1.

**FIGURE 2:**
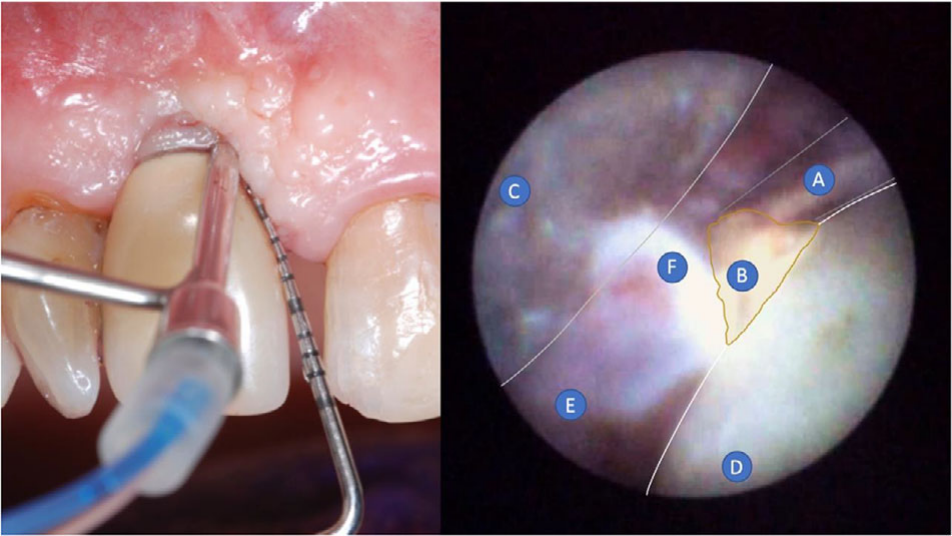
Clinical registration: On the left, the endoscope shield and the periodontal probe inserted below the mucosal margin of a tooth implant in position #2.1 can be seen. On the right, a snapshot of the endoscopic view. (A) Periodontal probe, (B) residual cement, (C) endoscopic shield, (D) prosthetic crown, (E) soft tissues, (F) granulation tissues.

The measurements were taken under endoscopic vision, positioning a millimeter periodontal probe (PCP UNC‐15) near the anatomical references mentioned above (Figure 2).

Once the collection of data for research purposes was completed, the deposit was removed. When possible, the removed deposit was recovered and evaluated under magnification to confirm the initial assignation.

### 2.2 Sample size determination

The 2009 study by Wilson Jr.^8^ reported that 80% of implants with cemented prostheses treated for peri‐implantitis (34 out of 42) exhibited subgingival cement residues. Using this percentage with a confidence interval width of 12.5% on each tail (exact confidence interval calculated with the binomial distribution) and at an alpha significance level of 5% yielded a minimum of 46 patients affected by mucositis or peri‐implantitis, with an expected number of patients whose implant site had cement residues equal to 37.^8^

### 2.3 Statistical analysis

Proportions for categorical variables and mean values ± standard deviations were used to describe the data. Associations between residual cement and the explicative clinical parameters were described by using stratified analysis; either the chi‐square test or the Mann‒Whitney *U* test, depending on the level of measurement of the variable, was used to evaluate the significance of the associations. The α level was a priori set at 0.05.

## 3 RESULTS

The demographic, behavioral, and clinical data of the 46 patients (one implant for each patient) included in the study are summarized in Table 1. All patients reported periodontitis at different severity levels.

**TABLE 1:** Patient characteristics.

Sex (%)	
M	54.3
F	45.7
Mean age, years (± SD)	49 (± 12)
Reasons for implant treatment (%)	
Periodontitis	58.7
Caries	28.3
Trauma	8.7
Agenesis	4.3
Smoking habit (%)	
>10 cigarettes/day	15.2
<10 cigarettes/day	10.9
Former smoker	15.2
Nonsmoker	58.7
Previous periodontitis (%)	100
Clinical picture (%)	
Peri‐implantitis	41.3
Mucositis	58.7
Time lapse between cementation and diagnosis, months (%)	
<6	10.9
6‐12	52.2
>12	37
Frequency of hygiene maintenance (%)	
<1/year	73.9
>1/year	26.1

No significant differences between patients with mucositis and patients with peri‐implantitis were observed except for hygienic maintenance periodicity: 100% of patients with peri‐implantitis performed hygienic maintenance less than once a year in comparison with 55.6% of patients with mucositis (*p* = 0.001).

Residual cement was detected in 37 of the 46 implants (80.4%). All cases identified endoscopically as cement and successfully recovered were confirmed to be cement after removal. No cement residue was misclassified as calculus and vice versa.

The data regarding the position of the implant in the arch and the position of the cement to the implant are shown in Table 2.

**TABLE 2:** Implant position and cement placement.

Implant position (*n*)	
Anterior maxilla	11
Anterior mandible	0
Posterior maxilla	18
Posterior mandible	17
Residual cement position (%)	
Lingual	59.5
Buccal	29.7
Distal	8.1
Mesial	2.7

Table 3 shows the associations between residual cement and clinical parameters. Residual cement was significantly more present when periodontitis was mild (*p* = 0.006) and when the implant was in the maxillary arch (both anterior and posterior) (*p* = 0.014) and was close to the significant limit when the diagnosis time was 6–12 and >12 months (*p* = 0.05).

**TABLE 3:** Associations between residual cement and clinical parameters.

	Cement present, *n* (%)	Cement absent, *n* (%)
Periodontal diagnosis		
Mild	13 (36.1)	0 (0)
Moderate	17 (47.2)	5 (55.6)
Advanced	6 (16.6)	4 (44.4)
Implant position		
Anterior maxilla	11 (29.7)	0 (0)
Posterior maxilla	16 (43.2)	2 (22.2)
Posterior mandible	10 (27)	7 (77.8)
Time in months		
<6	2 (5.4)	3 (33.3)
6–12	20 (54.1)	4 (44.4)
>12	15 (40.5)	2 (22.2)
Clinical picture		
Mucositis	24 (64.9)	3 (33.3)
Peri‐implantitis	13 (35.1)	6 (66.7)

The type of peri‐implant disease (mucositis and peri‐implantitis) did not appear to be directly associated with the presence of residual cement (chi‐square test; *p* = 0.085) but rather with its spatial position (*p* = 0.001).

Table 4 displays the distances between the reference points in the peri‐implant mucositis and peri‐implantitis frames.

**TABLE 4:** Mean distances ± standard deviation between reference points (min–max) in peri‐implant mucositis and peri‐implantitis frames.

Clinical picture	AC–IP distance (0.5–3 mm)	MM–PM distance (1–4 mm)	PM–IP distance (1.5–4 mm)
Mucositis	2.21 ± 0.29 mm	1.56 ± 0.52 mm	3.00 ± 0.44 mm
Peri‐implantitis	1.11 ± 0.42 mm	3.08 ± 0.49 mm	2.04 ± 0.52 mm
*p*	0.001	0.001	0.001

Abbreviations: AC, apical cement; IP, implant platform; MM, mucosal margin; PM, prosthetic margin.

Univariate analysis confirmed the significant influence of the position of the implant: All implants in the hemiarch anterior superior and 83% of implants in the posterior superior position presented residual cement (*p* = 0.03). Moreover, in the presence of residual cement, 89% of patients presented mucositis, and 63% presented peri‐implantitis (*p* = 0.04). Regarding the causes favoring the presence of residual cement, all patients with caries and 63% of patients with periodontitis presented cement (*p* = 0.03).

## 4 DISCUSSION

Peri‐implant disease is a crucial concern in implant dentistry, and cement retention for the prosthodontic frame has been suggested as a possible contributing factor. Residual cement around implant restorations seems to be capable of inducing inflammation and tissue destruction, leading to mucositis and peri‐implantitis.^4^, ^8^, ^11^, ^13^, ^19^

At present, the scientific position of this correlation is still pending, mainly because of the limited number of investigations focused on it.^20^

This endoscopic investigation of a cohort of patients with signs of peri‐implant disease revealed a considerable presence of submucosal cement excess. These data are consistent with the results of a previous similar study conducted by Wilson in 2009, suggesting that excess cement under the MM is not a rare event in cases of peri‐implant disease.^8^

The anatomical position of the debris detected can emphasize the real limitations of ordinary methods for detecting and removing excessive cement. Interestingly, 90% of the residues were located on the buccal or lingual sides, while only 10% were found in the proximal sites. The limited presence of interdental cement agrees with what was previously shown in a radiographic study.^21^

It is quite clear that tactile searching for submarginal excess cement is not a reliable method. On the other hand, radiographs can be effective only for interdental spaces, and the effectiveness can be further influenced by the radiopacity of various cements, reducing the sensitivity of this method.

As in this study, the use of a dental endoscope can easily overcome the limitations of conventional techniques, and it proved to be a reliable method for detecting and assessing submarginal residues. Several periodontal studies have already used this technology to evaluate submarginal deposits, confirming its clinical practicality, procedural atraumaticity, and reliability of observation.^8^, ^22^

The findings presented here are consistent with previous biological theories that propose a direct and/or indirect role of cement in the onset of peri‐implant disease.^23^, ^24^, ^25^

The present study provides new evidence suggesting that the spatial location of cement debris may influence the peri‐implant disease manifestation. From these findings, it can be speculated that the deeper the cement residue is, the greater the risk of peri‐implantitis compared to mucositis.

As reported in a specific review, the literature suggests that early detection of cement remnants (5 months) may lead to a higher detection rate of mucositis than peri‐implantitis.^11^ This is quite logical because mucositis is generally considered the early stage of the disease process that naturally precedes peri‐implantitis.

However, in the present investigation, where the most recent classification of peri‐implant disease was used, a considerable number of cases of peri‐implantitis were detected within the first year after implant loading. Furthermore, the time that elapsed between the occlusal load and the detection of residual cement did not show a correlation with the type of peri‐implant disease. Specifically, among the study variables, the only discernible difference between patients with mucositis and those with peri‐implantitis was the spatial location of the cement residue.

These considerations suggest that other aspects may play a role in disease manifestation.

It is plausible that the presence of peri‐implantitis in this study population may be connected to their prior history of periodontitis. To support this hypothesis, even in patients with previous mild periodontitis, the occurrence of peri‐implantitis was more prevalent in the presence of excess cement than in patients without it.

From a prosthetic point of view, referring to the distances between the MM–PM and PM–IP, it was observed that, in the presence of residual cement, the deeper and closer the PM is to the IP, the greater the probability of finding peri‐implantitis compared to mucositis.

This finding supports the literature on this topic, which recommends careful consideration of the placement of the PM.^4^, ^21^

As supported by the present study, without the use of an endoscope, the presence of excess cement can be challenging to diagnose, thus raising the question of whether cement‐retained restorations in general may be a risk factor for peri‐implantitis. In this context, some studies have not shown a significantly greater incidence or prevalence of peri‐implantitis between cement‐retained and screw‐retained implant crowns.^26^, ^27^, ^28^ This lack of correlation between cement‐retained restorations and peri‐implantitis should be carefully reconsidered under specific variables, some suggested by the present findings (such as the spatial positioning of the cement) and others by the literature (such as the type of cement used, removal techniques, etc.).^29^, ^30^, ^31^, ^32^

One possible explanation for these contrasting results is that the impact on peri‐implant tissue status is not influenced by the type of retention but rather by the presence of excess cement itself.

Excess cement may not be located in a way that causes disease, or there may be unknown protective factors that prevent or delay the development of peri‐implant diseases in certain patients. Additionally, it is possible that the occurrence of cement excess is not sufficiently common in the sample considered by these studies to significantly alter the proportions of biological complications between screw and cemented restorations.

Therefore, the selection of cement type and excess cement removal techniques are important factors to consider.^33^, ^34^ The main distinction among cements is between provisional and definitive cements. Provisional zinc oxide cements, free of eugenol, seem to offer advantages in this context as they are easily detectable in intraoral radiographs, are easily removable, allow for simpler removal of prosthetic restorations, and have the ability to reduce biofilm growth compared to that of definitive cements.^29^, ^35^, ^36^ However, the pathogenic potential of temporary residues is not known in detail, and there is a possibility that cement‐retained crowns using temporary cements and excess cement reduction techniques may loosen prematurely. When opting for the use of a definitive cement, greater attention should be given if the choice falls on resin cements as they are less prone to inhibit bacterial colonization and may be toxic to soft tissues due to the presence of free monomers.^29^, ^30^

Given the potential impact of excess cement on peri‐implant disease, clinicians must carefully manage the amount of cement used during implant restoration. Minimizing the amount of cement used and using specific strategies to achieve complete removal can help reduce the risk of peri‐implant disease. Numerous techniques for the removal of cement residues have been proposed in the scientific literature, but a solid comparative analysis between the various methods in the field of implantology is currently lacking.^31^, ^32^ Among these techniques, subgingival “crisscross shoeshine” flossing stands out as a method to be considered.^37^ However, it is crucial to pay attention during subgingival flossing to prevent the floss from tearing and thereby releasing potentially irritative residues that could affect the peri‐implant soft tissues.^38^, ^39^ Finally, handling cement residues seems crucial not only for its direct impact on soft tissues but also in terms of potential alteration to the titanium surface. Furthermore, mechanical removal should be carefully considered as it may produce fragments that can enter the soft tissues and lead to inflammation.^7^, ^40^

Further research is needed to better understand the mechanisms by which excess cement contributes to peri‐implant disease and to develop more effective cement removal protocols.

## 5 CONCLUSION

The results of this study support the hypothesis that there is a connection between cement residues and peri‐implant disease while further suggesting that the spatial position of cement residues may influence outcomes, with the most apical position potentially being correlated with peri‐implantitis.

## AUTHOR CONTRIBUTIONS

**Marco Montevecchi**: Contributed to conception; design; data acquisition; interpretation and drafted the manuscript. **Leoluca Valeriani**: Contributed to conception; design; interpretation; drafted the manuscript. **Maria Francesca Salvadori**: Contributed to conception; data acquisition; interpretation; drafted the manuscript. **Martina Stefanini**: Contributed to design; interpretation; critically revised the manuscript. **Giovanni Zucchelli**: Contributed to interpretation; design; critically revised the manuscript. All authors reviewed the final version and agreed to be accountable for all aspects of the work.

## CONFLICT OF INTEREST STATEMENT

The authors declare no funding or conflicts of interest for this article.

## FUNDING INFORMATION

The authors received no specific funding for this work.

## Data Availability

Data available on request from the authors.
